# Different Curcumin-Loaded Delivery Systems for Wound Healing Applications: A Comprehensive Review

**DOI:** 10.3390/pharmaceutics15010038

**Published:** 2022-12-22

**Authors:** Sarah A. Sideek, Hala B. El-Nassan, Ahmed R. Fares, Aliaa N. ElMeshad, Nermeen A. Elkasabgy

**Affiliations:** 1 Department of Pharmaceutics and Industrial Pharmacy, Faculty of Pharmacy, Cairo University, Cairo 11562, Egypt; 2 Pharmaceutical Organic Chemistry Department, Faculty of Pharmacy, Cairo University, Cairo 11562, Egypt; 3 Department of Pharmaceutics, Faculty of Pharmacy and Drug Technology, The Egyptian Chinese University, Cairo 11786, Egypt

**Keywords:** wound healing, curcumin, biomaterials, hydrogels, nanotechnology, wafers, sponges

## Abstract

Curcumin or turmeric is the active constituent of *Curcuma longa* L. It has marvelous medicinal applications in many diseases. When the skin integrity is compromised due to either acute or chronic wounds, the body initiates several steps leading to tissue healing and skin barrier function restoration. Curcumin has very strong antibacterial and antifungal activities with powerful wound healing ability owing to its antioxidant activity. Nevertheless, its poor oral bioavailability, low water solubility and rapid metabolism limit its medical use. Tailoring suitable drug delivery systems for carrying curcumin improves its pharmaceutical and pharmacological effects. This review summarizes the most recent reported curcumin-loaded delivery systems for wound healing purposes, chiefly hydrogels, films, wafers, and sponges. In addition, curcumin nanoformulations such as nanohydrogels, nanoparticles and nanofibers are also presented, which offer better solubility, bioavailability, and sustained release to augment curcumin wound healing effects through stimulating the different healing phases by the aid of the small carrier.

## 1. Introduction

Skin injuries (wounds) may be acute or chronic. An acute wound is a skin injury that occurs suddenly. It can cure in 2–3 months, depending on its depth and size and whether it is in the epidermis or dermis layers of the skin. Acute wounds can be resolved simply by covering the wound and depending on the self-healing mechanism of the body, while burns, infections, leg ulcers, and other chronic wounds are life-threatening because they do not automatically heal quickly [1,2,3,4].

Chronic wounds are superficial, partial or full-thickness skin loss that require intervention by secondary means to heal. Sometimes, it is crucial that they be addressed by an external treatment approach if a comorbidity of another illness [5].

Chronic wounds do not heal within an expected time frame, which is considered a serious clinical problem, owing to their increasing incidence and associated morbidity and mortality. A short time ago, the management of chronic wounds relied on outdated techniques such as reconstructive surgery or angioplasty and pharmaceutical interventions. The main cause of leg ulcers is arterial insufficiency, which often leads to failure of wound revascularization and finally limb amputation in arterial ulcer patients. They can be treated efficiently if reestablishment of arterial function occurs via revascularization, which is rather limited [6].

Although topical oxygen therapy was used as an adjunctive tool to improve tissue oxygenation, it was long criticized as having no role in this regard and nowadays many physicians choose not to use it on patients with chronic wounds [7].

Many factors can hinder the wound healing process, including the patients’ age and lifestyle (alcohol intake, smoking, and lack of exercise), all of which have a significant impact on the rate of wound closure (normally 10–14 days). Furthermore, patient health issues, such as excessive cholesterol, diabetes, peripheral artery disease, and hypothyroidism, might cause wound healing to be delayed [8].

The ability of living creatures to self-heal and face tissue damage is crucial for their survival. Tissue damage is defined as any change in the structure of tissue, whether it is hard or soft. Bones and teeth are examples of hard tissue, while ligaments, muscles, and tendons are examples of soft tissue that connect and support various body structures and organs. Chemical, mechanical, or even pathogenic factors can cause tissue injury [9]. Our bodies are programmed to undertake a self-healing procedure known as “tissue regeneration” to reverse tissue damage [10,11]. Tissue/organ transplantation is the only option when the damage is so severe that the body’s natural self-healing process cannot keep up with cellular death or when the cells in the injured tissue are nonreplicating. However, there are significant drawbacks to transplants, including a limited number of donors and the possibility of transplant rejection. Due to these restrictions, researchers began looking for a more viable and interdisciplinary strategy that might be used to replace traditional therapy and address organ shortage [12]. To restore normal tissue/organ function, one suggestion called for the regeneration of new tissues in place of the damaged ones. This was the first time, in 1933, that the term “tissue engineering” was used [5].

**Figure 1:**
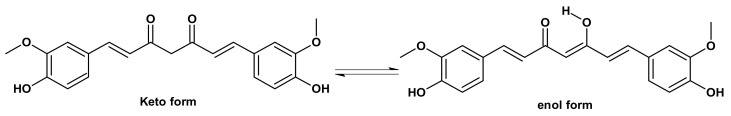
Keto–enol tautomerism of curcumin.

Turmeric is a spice isolated from the rhizomes of *Curcuma longa* L. and is widely consumed in Southeast Asia. It is used in traditional medicine in China and India and is known as the “golden cure,” owing to its wide biological activities [13]. The main constituent of turmeric is the polyphenol curcumin [(1E,6E)-1,7-bis(4-hydroxy-3-methoxyphenyl)-1,6-heptadien-3,5-dione, also known as diferuloylmethane (Figure 1). Curcumin displays many biological activities, including antibacterial, antifungal, antiviral, antioxidant, anti-inflammatory, and anticancer activities [14,15,16].

The wound healing capacity [17] of curcumin is attributed to its antioxidant, antibacterial and anti-inflammatory properties [18,19,20]. Curcumin is known to influence the inflammatory and proliferative phases in wound healing [21]. Additionally, it can induce and enhance angiogenesis during the wound healing process [22].

Curcumin is safe to be ingested orally up to 12 g/day with no sign of toxicity or adverse effects. Nevertheless, its clinical use is limited by its poor bioavailability, rapid metabolism and chemical instability in alkaline medium [23,24,25,26,27]. This is attributed to the keto–enol tautomerism of curcumin that hinders its absorption (Figure 1). At low pH (3–7), curcumin is present in the keto form, while at high intestinal pH the enol form predominates [28]. It is worth mentioning that the enol form acts as electron donor and is responsible together with the phenolic group for the antioxidant effect of curcumin [16]. Curcumin is rapidly metabolized by aldo–keto reductases that target the β-diketone moiety of curcumin [15,27]. Curcumin is chemically stable at pH 1–6; however, it is insoluble in water at this pH. On the other hand, curcumin is rapidly degraded in phosphate buffer at pH 7.4 to yield many degradation products, including vanillin, vanillic acid, ferulic aldehyde, ferulic acid, feruloylmethane [28] and trans-6-(4′-hydroxy-3′-methoxyphenyl)-2,4-dioxo-5-hexenal.

To get the maximum benefits from this miracle molecule, new technologies and strategies were introduced, such as formulating curcumin nanoformulations that offered a go-to option for better wound healing purposes [29].

## 2. Wound Healing

A wound can be limited to the epidermal layer, which heals via reepithelialization without the need for a skin graft, or a full-thickness wound (FTW), which requires both epidermis and dermis to heal. In this circumstance, complete reepithelialization of the skin either takes longer, resulting in substantial impairment, or necessitates the use of skin tissue-regenerating product to speed up the process of wound healing. The main issue with open FTW is the susceptibility to microbial infections, which activates the immune system and increases the neutrophil production. Even endogenous antioxidants such as superoxide dismutase, catalase, and glutathione, which serve as the first line of defense against free radicals, are unable to neutralize the microbial attack in this condition. As a result, the fibroblasts—the major cells responsible for collagen synthesis and extracellular matrix (ECM) development—suffer from oxidative damage. This condition, however, can be alleviated by administering both antioxidant and antibacterial agents [30]. Wounds may be classified simply as acute and chronic wounds. Chronic wounds are the most resistant to treatment modalities, especially if accompanied by other illness.

Chronic wounds have a bad repair process, leading to the wounds not healing within 3 months. The most common causes of chronic wounds are diabetes, aging, paraplegia and obesity, which put a financial burden on the health-care sector. Among the chronic wounds are nonhealing pressure ulcers (bed sores) and venous and diabetic foot ulcers [31].

Chronic wounds are always associated with chronic inflammation that is characterized ny the occurrence of significant neutrophil infiltration coupled with a rise in reactive oxygen species (ROS) levels due to the diminished macrophage phagocytic ability [30].

There are many factors that could prevent or promote wound healing progress, such as local wound cleanliness, which may be a key component in preventing subsequent infection and suppressing potential triggers of an abnormal immune response [32].

Some diseases can definitely aggravate the wound healing pathway progression, such as diabetes mellitus (DM) and paraplegia.

DM is a significant risk factor for the development and persistence of chronic wounds. In diabetics, neuropathy is a possible cause of delayed wound healing. Diabetics have fewer nerves in their epidermis and dermis due to their inability to regenerate nerves. Moreover, the formation of new vessels is difficult due to chronic hyperglycemia, which limits oxygen and nutrients access to the wound site. Because of the previous reasons in addition to persistent inflammation, infections, and degradation or reduced expression of growth factors, the ordinary treatment strategy (glycemic control, continuous wound debridement and infection control) may not be enough, and therefore external supporters are needed to promote diabetic wound healing. Currently, several bioactive molecules, i.e., growth factors, genes, peptides, and stem cells, as well as nonbioactive materials such as metal ions and oxygen, are applied to promote wound healing in cases of DM [33,34].

Paraplegia is considered another risk factor for the occurrence of chronic wounds. Paraplegic patients with neurological impairment due to tissue denervation and lack of tissue perfusion owing to a state of hypotension are characterized by this illness. All of these comorbidities prevent essential moieties from reaching the wound. The denervated tissue is unable to produce an inflammatory response, which results in a shortage of oxygen delivery to the wound, hence delaying many stages of the wound healing process, including angiogenesis. All these risk factors and more have a significant impact on the stages of wound healing. Wound healing occurs over four stages, and any interruption for one or more of these stages will definitely hinder the healing progress [35].

### Stages of Wound Healing

After a tissue injury, wound healing, a normal biological process in the human body, begins to repair and protect the body from further damage caused by infection, blood loss, and other issues. For a wound to successfully heal, four highly programmed overlapping phases, namely, hemostasis, inflammation, proliferation, and remodeling, must occur in this order and within a specific time frame, and any interference in one or more stage will negatively influence the healing mechanism [36].

Hemostasis’s main function is the protection of the vascular system and prevention of corresponding loss of organ function triggered by vasoconstriction in reaction to the damage as a way to prevent blood and fluids loss. Vasoconstriction is then followed by platelet invasion from the surrounding vessels to form a plug. The inflammatory phase goal is to create an immunological barrier against the incoming bacterial contamination and eradicate germs that are introduced into the wound because of the tissue damage. Briefly, leukocytes arrive at the site of injury immediately after the initial hemostatic reaction, followed by the deposition of several immune cells [37].

**Figure 2:**
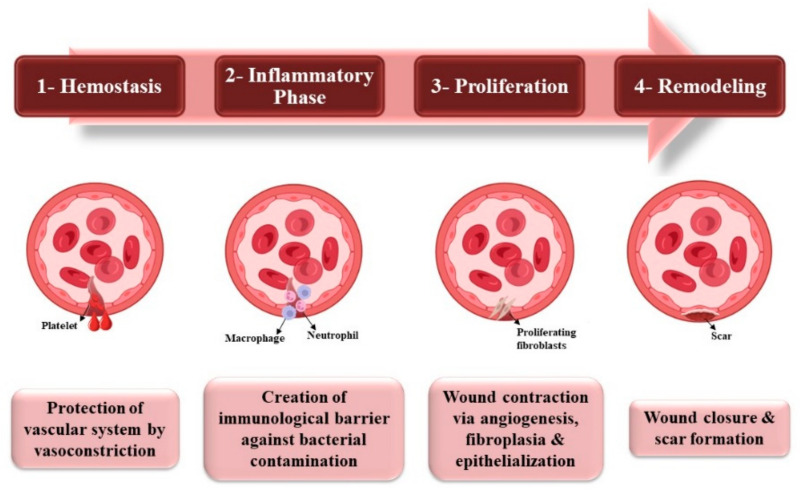
A schematic representation illustrating the different stages of wound healing. (Created with BioRender.com; accessed on 12 December 2022).

The proliferative phase starts with the re-creation of an epithelial barrier by contraction of the wound via different processes including angiogenesis, fibroplasia and epithelialization. The reestablishment of functional microvasculature and the elimination of damaging bacteria is followed by remodeling. Dermal and epidermal cell regeneration can happen, resulting in wound closure and scar formation. The remodeling phase is the final phase, characterized by regression of several freshly formed capillaries and physical contraction mediated by fibroblasts. ECM remodeling of architecturally normal tissue is a critical feature of this phase [38]. Chronic wounds can develop if any of the four stages of wound healing are not completed. In many instances, microbial infection is the big obstacle to wound healing. Macrophages and neutrophils are the initial line of defense against invading microorganisms when the tissue is wounded [35,39]. The steps for wound healing are illustrated in Figure 2.

## 3. Curcumin-Loaded Delivery Systems for Wound Healing

The marvelous healing power of curcumin is long known and its role in the improvement of several types of topical skin wounds is due to its antioxidant activity, improvement of the production of granulation tissue and new vascularization and increasing the process of reepithelialization of wound damage, but unfortunately all of those useful applications are limited by its bad solubility and stability. Tailoring suitable drug delivery systems for carrying curcumin improves its pharmaceutical and pharmacological effects. In brief, curcumin is a natural molecule separated from the rhizomes of *Curcuma longa* and is used topically for many wound healing applications [40].

Among the drug delivery systems designed for wound healing, hydrogels are the most widely used due to their similarity to the native extracellular matrix and capability to provide a humid environment [41].

**Figure 3:**
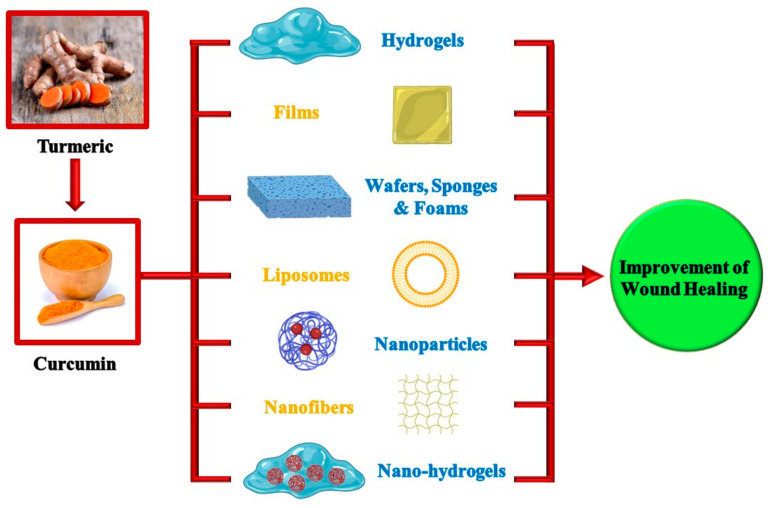
A collective illustrative diagram representing different dosage forms used for delivering curcumin at the wound site for better wound healing. (Created with BioRender.com; accessed on 12 December 2022).

A brief summary of the most recent work published on the use of curcumin-loaded drug delivery systems in wound healing is given below. Figure 3 illustrates the various dosage forms used for delivering the curcumin at the wound site.

### 3.1. Hydrogels

The literature is rich with many different studies exploiting the applications of hydrogels for wound healing [41,42,43]. Polymeric hydrogels have been extensively applied in biomedicine and TE because of their unique ECM-mimicking tunable properties of tissue, such as water retention of ten to thousand times their equivalent weight, biocompatibility, biodegradability, and their cell support during tissue regeneration [41].

Polymeric hydrogels can be classified according to the method involved in their formation into noncovalent (physical) and covalent hydrogels (chemical) [44]. Physical or chemical cross-linking interactions can be used to generate an insoluble three-dimensional network from any hydrophilic polymer.

#### 3.1.1. Noncovalent Hydrogels (Physical Hydrogels)

A noncovalent interaction is different from a covalent one in that it includes more scattered electromagnetic interactions between molecules or within a molecule rather than exchanging electrons. There are numerous subcategories of noncovalent interactions, including electrostatic and van der Waals forces and hydrophobic effects [45,46].

These interactions have a significant impact on drug and material design. Dynamic noncovalent interactions have been exploited in designing hydrogel systems. This has revealed a variety of truly amazing hydrogel properties, such as self-healing, thermos responsiveness and/or pH responsiveness, self-recovery, shape memory, remoldability and many other applications. The influence of relatively weak internal attractive interactions such as hydrogen bonding, dipole–dipole forces, and hydrophobic interactions make the preparation of the hydrogels covered in this category feasible [47,48,49].

Different types of noncovalently bound hydrogels include thermally annealing [50], pH-dependent [47,51], ionically cross-linked [52] and self-assembling peptide-based hydrogels [53].

#### 3.1.2. Covalent Hydrogels

Covalent hydrogels have been extensively utilized in regenerative medicine. Covalent cross-links are irreversible, resulting in stiffer and more mechanically stable substances [48]. Covalent hydrogels can be formulated into more mechanically robust scaffolds due to their higher stability [54]. Formerly, these developed substances were delivered by invasive surgical interventions; however, novel ways of formation, such as photo-induced radical polymerization or delayed bonding through slow kinetic reactions, have enabled their delivery to the required site via noninvasive injection [55].The stronger binding forces within the covalent hydrogels hinder their dilution with the surrounding fluid or their diffusion away from the injection site, which results in an efficient and long-lasting effect [48].

Different examples in the literature elaborate the preparation of curcumin-loaded hydrogels for wound healing purposes. In one study, two types of curcumin-loaded hydrogels were prepared: single-loaded hydrogels composed of chitosan and Pluronic 123 and curcumin and dual-loaded hydrogel consisting of chitosan, Pluronic 123, gelatin, and curcumin. The results showed that single-loaded hydrogel showed a rising in swelling rate from 48 h to 400 h, where it reached its maximum level (268.51 ± 0.16%) after 432 h. On the other hand, the marvelous water absorption capabilities of gelatin in the dual-loaded hydrogel raised the swelling behavior more than 1.2 times compared to the single-loaded hydrogel. The results of the release profiles of curcumin from single- and dual-loaded hydrogels showed initial burst release in the first 2 h, followed by a steady sustained phase up to 50 h. However, the dual-loaded hydrogel showed more sustained drug release due to the denser structure of hydrogel because of the gelatin presence. In vivo tests were conducted on 16 mice. Their backs were shaved and they were randomized into four groups: untreated, unmedicated single-loaded hydrogel, medicated single-loaded hydrogel, and medicated dual-loaded hydrogel. An FTW was made on the shaved backs and wound area changes were observed after the application of different hydrogel samples. Images of the wound surface were captured and the area of the wound was investigated to assess the rate of wound healing. The results showed that the healing rate determined by the area of wound was more than 98% for the three types of hydrogels compared to the untreated wounds [56].

### 3.2. Films

Films are one of the popular techniques for wound healing. They are extremely flexible, transparent and adhesive. Although they have negligible capacity to absorb fluid, they are able to absorb little amounts of fluid by a moisture vapor transpiration, which means that such films are not suitable for wounds that produce huge amount of exudate [57].

Gopinath et al. succeeded in designing curcumin-loaded films made from collagen. The in vivo results after the application of the medicated films on induced wounds in rats were compared to the unmedicated hydrogel along with the control. On the 7th day following wound treatment, results revealed great increase in the amount of neutrophils along with proliferating fibroblasts and macrophages in the group treated with medicated hydrogels compared to the other two group, highlighting the synergistic roles of curcumin as well as the other formulation ingredients in enhancing the healing process [58].

### 3.3. Wafers

Wafers are another example of dosage forms used for treating wounds. Adel et al. presented a simple way for the formation of curcumin-loaded freeze-dried chi-tosan-based wafers without using harmful cross linkers and ensuring the formation of highly porous matrices. Composite wafers were fabricated by adding sodium hyaluronate to enhance the porosity. The developed wafer formulation significantly reduced the levels of tumor necrosis factor α and the levels of interleukin 6 compared to the unmedicated wafer formulation as well as the free drug. Generally, the reduction in cytokine levels could be due to the dual anti-inflammatory effects of curcumin and chitosan. Microbiological assays proved the excellence of the chosen curcumin wafers against free curcumin in bacterial growth inhibition on *Staphylococcus epidermidis* and *S. aureus* [59]. Further investigations on animal models are required to assess the efficacy of the developed curcumin-loaded wafers for wound healing.

### 3.4. Sponges and Foams

Medicated sponges are a new and promising technology that offers targeted and controlled drug delivery. They are based on polymeric spheres that can entrap a wide variety of materials and then be incorporated into a formulated product such as gels, lotions, creams, ointments, liquid or powders. This novel technique results in fewer side effects, better stability, and the advantage of targeting the drug delivery of either lipophilic or hydrophilic drugs [60].

Nguyen et al. proposed the formation of curcumin-loaded composite sponges made of chitosan and gelatin mixed in various ratios. The results revealed that the combination of curcumin, chitosan and gelatin could significantly improve the wound healing capability in comparison to the sponges lacking the addition of curcumin. The cytocompatibility tests on L929 fibroblast cells indicated the safety of the investigated curcumin-loaded sponges. Wound closure was measured in the induced wounds in albino rabbits at predetermined time points and the results were compared for the treated (medicated sponge; chitosan:gelatin mixed in ratio 3:1) and the untreated groups (unmedicated sponge). After examining the wound area with a digital camera, results revealed that there was a significant increase in wound closure on the 12nd and 15th days posttreatment for the treated group compared to the untreated one. These results proved the wound healing ability of the formulation, making it an ideal choice for many wound dressing applications [61].

Hegge et al. formulated alginate-gelling foams encapsulating curcumin for wound healing applications, especially designed for highly exuding wounds because of the high water absorptivity of the formed polymeric foams at physiological pH and sustained drug release. The preparation method depended on the cross-linking reaction between sodium alginate and calcium ions [62] utilizing gluconic acid *δ*-lactone as a pH modifier and hydroxypropyl methylcellulose as foaming agent. In this study, the authors merged the use of the antimicrobial photodynamic therapy on highly infected wounds, where bacterial infections could significantly interfere with the normal wound healing mechanisms of the human body. Cultures of Gram-positive bacteria such as *Echoli faecalis* were treated with the medicated foams and the results showed the photodynamic inactivation of the exposed cultures, where >6 log^10^ reduction of viable cells was noticed. On the other hand, no decrease in the bacterial viability was observed in any of the treated cultures in the absence of light [63].

### 3.5. Application of Different Nanotechnology-Based Approaches in Wound Healing

The most important treatment techniques for improving the medical and pharmaceutical applications include the application of nanotechnology [64,65]. Nanotechnology-based diagnostics and treatment techniques offer a promising pathway to target the whole cycle of the wound healing process with the unique properties of nanocarriers, such as the higher surface area to volume ratio, which increases the chances of tissues penetration [31,66]. Nanocarriers are perfect for inducing cell-to-cell contacts, cell proliferation, vascularization, cell signaling, and biomolecule synthesis, all of which are required for efficient wound healing. Additionally, nanocarriers have the ability to deliver one or more therapeutic molecules, including growth factors, nucleic acids, antibiotics, and antioxidants, to the target area in a sustained way [67].

The application of nanotechnology for the preparation of different wound healing dosage forms offers a superior approach to fasten the healing of acute and chronic wounds through stimulating the different healing phases by the aid of the small carrier. Different examples of nanocarriers used for wound healing include liposomes, nanoparticles, nanofibers and nanohydrogels [68,69,70,71,72,73,74].

#### 3.5.1. Liposomes

Curcumin is an efficient wound healing agent in burn therapy; however, it must be applied topically due to its low bioavailability. Dermal delivery systems based on liposomal nanocarriers were developed by Kianvash et al. to treat rats with second-degree burns using curcumin-propylene glycol liposomes. The wound healing efficacy of the developed liposomal formulation was investigated by comparing the results of four samples on induced second-degree burns in male Wistar rats. The four groups were control (plain liposomes), silver sulfadiazine cream (1% *w*/*w*), curcumin–propylene glycol liposomes (0.3% *w*/*w*) and free curcumin 0.3% groups. The curcumin–propylene glycol liposome group showed the highest wound recovery (*p* < 0.001) of 95.8 ± 2.28% where the treated wounds appeared thin, light pink to yellow, with exudate surprisingly fully controlled on the 7th day. The selected formulation showed no cytotoxic effects on human dermal fibroblasts, indicating the that the selected dose was a safe one [75].

#### 3.5.2. Nanoparticles

The wound healing effect of curcumin was reinforced by the preparation of polymeric nanoparticles using polymers such as poly(lactic-co-glycolic acid). The encapsulation of curcumin protected the drug from light degradation, enhanced water solubility, and sustained curcumin release over a period of 8 days, which in turn resulted in accelerating angiogenesis and wound healing processes. The success of the prepared nanoparticles was obvious when the drug-loaded nanoparticles managed to accelerate the wound healing, as was proved in an FTW (excisional wound) mouse model. The wound areas were visualized over a period of 16 days and the results revealed that on the 10th day post-procedure, curcumin-loaded nanoparticles showed an accelerated rate of wound healing compared to that of the control and the other (free curcumin and unmedicated nanoparticles without curcumin) groups. Complete wound closure was observed with curcumin-loaded nanoparticles, whereas the other groups showed wound recovery of about 75% [76].

Alqahtani et al. reported the application of curcumin-loaded lignin nanoparticles in wound healing. Both curcumin-free as well as -loaded nanoparticles were found to be biocompatible with strong in vitro antibacterial activity against Gram-positive pathogens, especially *Staphylococcus*. Wounded rats treated with curcumin-loaded lignin presented greater wound closure compared to the control group. After 12 days, nearly full wound contraction was observed in treated groups versus approximately 43% wound contraction in the untreated control group. Moreover, curcumin-loaded lignin nanoparticle-treated wounds displayed enhanced granulation tissue formation and more collagen deposition [77].

One study examined the synergistic effect of polymers from different sources (natural, synthetic and semisynthetic) with curcumin for the preparation of nanoparticles for wound healing applications. In the study, chitosan, carboxy methyl cellulose, and polylactic co-glycolic acid were used as natural, semisynthetic and synthetic sources. Based on the source of the polymer, the fabrication method differed, where the ionotropic gelation method was applied for chitosan and carboxymethyl cellulose, while the double-emulsion solvent evaporation method was conducted for polylactic co-glycolic acid. The in vivo model, Wistar male rats with an FTW on their backs, revealed the success of curcumin-loaded chitosan nanoparticles in enhancing the healing process, which might be due to the synergistic effect of the combination of both curcumin and chitosan. Also, results showed that this combination led to the reduction in curcumin dose for efficient wound healing [78]. Using natural polymers for the preparation of nanoparticles is considered a safe and cost-effective strategy. This nanocarrier can be tailored for the delivery of other water-insoluble drugs.

Another study highlighted the substantial benefits of nanoparticles in improving the solubility of curcumin and the percent of its loading, hence the therapeutic efficacy. In brief, curcumin-loaded nanoparticles (5 mg/mL) were prepared and loaded into methoxy poly(ethylene glycol)–graft–chitosan composite film for wound healing purposes. By investigating the wound healing ability of the fabricated films on FTW developed in rats, results showed that the antioxidant activity of curcumin did not change after its incorporation in the nanoparticles firstly, then into the film compared to the unmodified curcumin. Histopathological results revealed the superior improvement in the healing process in the group treated with the medicated film compared to the unmedicated film, where the epithelization process was faster and the formed collagen was compact and well developed [79]. The obtained results suggested a promising carrier for insoluble drugs’ wound healing purposes.

#### 3.5.3. Nanofibers

Nanofibers are a special type of nanomaterials that have their own qualities and characteristics. Nanofibers have a diameter of around 10–100 nm as well as a large specific surface area. They are made via electrospinning, which involves using electric force to generate nanometric fibers from various polymer solutions. This method is inexpensive and easy. These fibers can form a highly porous mesh with fine interconnectivity [80,81].

Nanofibers have the same size as natural ECM fibers, and can be simply designed to simulate the stiffness of a wide range of soft tissue [74].

Curcumin-loaded nanofibers are an attractive and promising strategy to treat FTW, but the main obstacle is the difficult solubilization due to its hydrophobic nature. Shefa et al. overcame this drawback by formulating the physical cross-linkage of nanofibers using the freeze–thaw technique. Pluronic F-127 was added to solubilize the drug, polyvinyl alcohol to enhance the gelation properties and TEMPO-oxidized cellulose nanofibers were used to enhance the porosity. The created hydrogel was a biocompatible and biodegradable system for curcumin delivery. The results showed that the wound healing potential of the developed nanofibers after examining the control and curcumin-loaded hydrogel one-week post-application of the samples on FTW in a rat model that the wound closure in the treated group was significantly higher than the untreated group: 29.9 ± 1.7% versus 8.3 ± 1.13%, respectively. While the wound size in all groups decreased as time passed, the wound size of the curcumin-loaded hydrogel groups was smaller than the others, and the wounds had almost closed by 2 weeks [82].

Dhurai et al. reported the design of curcumin-loaded chitosan/poly(lactic acid) nanofibers using electrospinning and examining the in vivo wound healing activity on excision and incision wounds created in a rat model for a total of 21 days. Chitosan’s role was the wettability gradient, which may directly enhance blood clot stabilization and consequently wound healing. The examination revealed the significant reduction of wound area when compared to untreated control. Significant wound area reduction was noticed where the untreated control and treated groups showed 85.37% and 95.51% reduction, respectively. This improvement might be attributed to the hemostatic role of chitosan, which greatly controlled the bleeding and aided in speeding-up the epithelialization rate. In the same context, curcumin reduced the levels of ROS by its excellent scavenging properties of the free radicals produced in the wound [83].

In the same context, Moradkhannejhad et al. [84] followed the same idea of Dhurai et al. [83] but with some minor modifications. Curcumin-loaded poly(lactic acid) nanofibers were formed by electrospinning and the hydrophilicity of nanofibers was improved by the addition of poly(ethylene glycol) with molecular weight of 1500 in different concentrations (0, 5, 10, 15, and 20 wt% relative to the poly(lactic acid) content). The cell proliferation capability of the designed curcumin-loaded nanofibers against MG-63 cells was investigated after 24 h of cell incubation with the formulation. It was found that better cellular attachment and proliferation were observed with nanofibers prepared using the highest concentration of poly(ethylene glycol). This might be attributed to the increased hydrophilicity and wettability of designed nanofibers compared to the nanofibers lacking the addition of poly(ethylene glycol). Hydrophilic nanofibers could provide a suitable media for cell growth, attachment and proliferation and thus could greatly enhance the wound healing applications [84].

The use of graphene-based dressings together with curcumin is another interesting approach for wound healing applications owing to their antimicrobial characters. One study developed graphene oxide–TiO_2_–curcumin-integrated cellulose acetate nanofibers for wound healing applications using simple electrospinning. The developed nanofibers possessed higher tensile strength as well as better antibacterial activity than pure cellulose acetate nanofibers. Moreover, the developed nanofibers showed better hemocompatibility. Cell studies on fibroblasts showed better cellular proliferation, which might be attributed to enhanced re-epithelialization and antibacterial properties [85].

Further in vivo studies are required to ensure the efficacy of developed curcumin-loaded nanofibers and dressings.

### 3.6. Nanohydrogels

Merging the benefits of both conventional dosage forms with that of nanotechnology improved the performance and overcame the limitations (poor integration with native human skin tissue, low cellular uptake of genetic material used as aiding therapy in wound healing and unsuitable microenvironment for wound healing) of each of them when used solely [73,86].

This linkage has been proposed to be the cornerstone for the future applications of wound healing and TE techniques, where the incorporation of the nanomaterials in hydrogels can improve the performance and the therapeutic effect of drugs, making the wound dressing more practical [73,87,88].

Nanohydrogels are one of the most important drug delivery systems for both time-controlled release and targeted delivery of drugs. This system combines the marvelous advantages of both the hydrogels and nanocarriers in one system [89]. Nanohydrogels can hold drugs, macromolecules and are capable of responding to external stimuli. This makes them suitable for wide range of applications [90].

Nanohydrogels are characterized by different properties that make them unique for wound healing purposes. Firstly, controlled drug release, where the loaded nanocarriers control the drug release through its matrix by tailoring their structure, particle size, and manufacturing conditions. Moreover, the released drug molecules from the nanocarriers need to pass through the hydrogel matrix through diffusion or swelling or chemically controlled mechanisms. Secondly, the stimuli-responsive properties of some polymers can aid in the preparation of drug-loaded nanohydrogels with controlled and targeted drug release in response to certain stimuli (temperature, pH, light, etc.) [88,91,92,93,94,95,96].

This was proved by the proposed model of Han et al. where they made use of the near-infrared responsive hydrogel (type of stimuli-responsive hydrogel), which can be effectively controlled by changing the radiation intensity and exposure time to light source, where polydopamine nanoparticles were introduced into a poly(N-isopropylacrylamide) network to form a (polydopamine nanoparticles/poly(N-isopropylacrylamide) hydrogel with near-infrared capability used for self-healing applications with increased cell/tissue adhesiveness. The near-infrared-assisted healing was proved by in vivo FTW experiments, demonstrating that (polydopamine nanoparticles/poly(N-isopropylacrylamide) had a synergistic effect on accelerating wound healing [97]. Thirdly, site-specific drug delivery. Nanohydrogels can entrap the loaded nanocarriers within 3D polymer matrices, allowing better local drug delivery. Various nanohydrogels have been developed to target the drugs to the spinal cord [98,99], the eye [100] and the skin [38].

Pathan et al. reported the formulation of curcumin-loaded fish scale collagen-hydroxypropyl methyl cellulose nanohydrogel for wound healing applications. By examining the in vivo results it was revealed that the healing of wounds in Wistar rats showed better response with medicated formulation compared to blank, where a significant increase (*p* < 0.05) in contraction value (95.42 ± 12.20%) on the 20th day was obtained compared to other formulations, where contraction values were less than 68.45 ±10.20%. This might be due to the improved granulation tissue formation and re-epithelialization, which led to a reduction in wound size [101].

Another study reported the successful preparation of composite dressing made by mixing nanosilver nanohydrogel, *Aloe vera*, and curcumin with a gel system composed of polyvinyl alcohol, polyethylene oxide and carboxymethyl cellulose. This blend was later coated onto hydrolyzed poly ester fabric to fabricate a wound dressing with antimicrobial, infection control and wound healing nature. The antibacterial analysis of the fabricated curcumin-loaded fabrics on *Escherichia coli* and *Staphylococcus aureus* showed a massive bacterial reduction of over 80%, indicating the synergistic roles of both curcumin and nanosilver nanohydrogel as antibacterial agents. Moreover, the treated wounds showed faster healing with minimum scarring indicating the efficacy of the used fabric due to speeding up the re-epithelialization rates [102].

Liposomes are considered the most abundant nanocarriers due to their biocompatibility and passive targeting abilities. However, some disadvantages limit their use, such as difficulties in maintaining the drug concentration in the diseased area, while merging liposomes within hydrogel matrix improves their clinical application [103].

Surgical wound healing rate is a key factor for chronic wounds, and quickening this slow rate will definitely influence wound closure and healing time. Cardoso-Daodu et al. succeeded in formulating lysine–collagen hydrogel-containing curcumin-loaded liposomes. In vivo results on 15 male Wistar rats, weighing 350–400 g, showed that after 3 days of the surgical procedure, the nanohydrogel formulation had the highest percentage wound contraction and closure of 79.25% compared to the control group, which showed complete closure by day 14 post-surgery with obvious scarification [104].

Metal nanocarriers and hydrogels have recently been combined to create composite materials with novel or better characteristics. These hybrid materials permit biomedical applications in a variety of ways by accurately manipulating the composition, configuration, and interactions of their elements [105]. Combination of metal nanocarriers such as silver [106], gold and copper nanocarriers with a hydrogel matrix could efficiently inhibit bacterial growth and at the same time fasten the healing process of a wound [107].

Table 1 lists different studies highlighting the design of different curcumin-loaded dosage forms intended for wound healing purposes.

## 4. New Technologies

Bioprinting is a new type of technology that allows living cells, biomaterials, and growth factors to be printed in complicated 3D structures. Bioprinting uses a computer-controlled 3D printer to construct 3D structures in a layer-by-layer printing process, which enables a great degree of flexibility and repeatability [96]. One promising example is the ex vivo approach where the stem cells are extracted from the donor and seeded either on or within a scaffold. After that, the cell-laden scaffold is triggered in a suitable environment in bioreactors by certain signaling cues that promote the proliferation and thus healing of the desired tissue type [12,123]. Because not all types of cells can perform repair after damage, such as cardiac muscle cells, stem cells have the ability to proliferate into the desired cell types [124].

**Figure 4:**
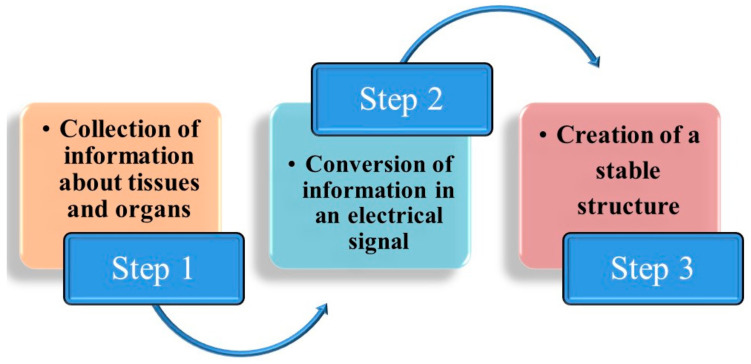
Illustrative diagram showing the steps for the 3D bioprinting.

Three processes are usually included in bioprinting. Firstly, information about tissues and organs is collected for model designation and material selection; secondly, the information is converted into an electrical signal that controls the printer to print the tissues; and thirdly, a stable structure is created [125]. Figure 4 displays the steps required for 3D bioprinting.

It is well known that cell viability can be influenced by several factors, including the bioprinting technique used, shear stress (influenced by the technique pressure and hydrogel viscosity) [126]. In addition, resolution of light-assisted bioprinting may vary according to several factors, such as density and viscosity of the inserted biological material [127], laser pulse intensity, printing speed and structural organization).

The novel bioprinting process has been viewed as a cornerstone for future applications of organ transplants. However, there will always be a demand on versatile new fabrication techniques that may control the growing organ demand, shortage and poor tissue healing as well as provide a highly precise method for cell patterning and architecture at the micrometer scale for biomedical tissue engineering applications and wound healing [128].

One study discussed the preparation of 3D printed curcumin-loaded gelatin methacryloyl hydrogels which were stem cells-laden. Results showed that the developed hydrogels prepared using 10% gelatin methacryloyl formed biocompatible hydrogels with good printability. The selected hydrogel was applied to diabetic wounds on nude mice and the results were promising. The loaded curcumin mitigated ROS production and enhanced the healing process. The loaded stem cells augmented the role of curcumin in improving the wound healing properties [129]. Clinical studies should be carried out for more investigations.

Advances in 3D bioprinting have paved the way for better wound healing applications. However, the high cost of the process limits its use. Several attempts have been made by scientists to reduce the required costs. Among these attempts was the conversion of commercially available 3D printers into a low-cost 3D bioprinter using cell-laden customized bioink based on biocompatible polymers such as alginate and gelatin mixtures. By adjusting the proportions of each component in the custom-made bioink, the rheological properties of the bioink can be tuned for supporting the proliferation of the cell lines, with higher accuracy and better resolution of the printed constructs [130].

## 5. Conclusions and Future Perspectives

Over the years, chemical and pharmaceutical companies have manufactured several curcumin products for the treatment of multiple diseases. Based on the studies reported in the preceding sections, it can be concluded that curcumin has a strong modulating effect on the wound healing process. Curcumin accelerates the rate of wound healing by affecting all the wound healing phases owing to its antibacterial, antioxidant and anti-inflammatory properties. Nevertheless, its poor oral bioavailability, low water solubility and rapid metabolism limit its medical use, so tailoring new curcumin delivery systems was adopted. Various topical formulations of curcumin-including nanoarchitectures have been developed and evaluated that offer better solubility, bioavailability, and sustained release to augment curcumin wound healing effects.

Hydrogels, flexible film, wafers, and sponges loaded with curcumin all showed improved wound healing capabilities and better antibacterial effects when combined with an antimicrobial therapy for infected wounds. Nanoparticles encapsulating curcumin showed better stability to light, solubility and sustained release for days, resulting in accelerated angiogenesis and wound healing processes. Combining the advantages of both hydrogels and nanocarriers in the nanohydrogels has been proposed to be the cornerstone of future applications of wound healing and tissue engineering techniques, as it improves their performance and makes wound dressing more practical.

More efforts should be made by scientists to find new modalities for wider applications of 3D bioprinting in the wound healing field. New cost-effective strategies should be introduced.

Although there is plenty of research that has been done on curcumin formulations, most of the studies reported are in the preclinical phase, though with promising results. To be able to maximize the benefits of curcumin as a wound healing aid, these formulations need to reach clinical trials. Also, the scaling up and large-scale industrial production of curcumin formulations for wound healing should be addressed and covered in future work plans.

## Figures and Tables

**Table 1:** Different examples of curcumin-loaded dosage forms for wound healing purposes.

Formulation Type	Polymers Used	Key Findings	Reference
**Hydrogel membrane**	Chitosan and sodium alginate	The in vivo testing on FTW induced in rats presented enhanced reepithelialization (75 ± 2.3%) within 14 days post treatment compared to the wounds covered with gauze. The formed tissues showed well-defined epidermis and stratum corneum with higher % of collagen.	[108]
**Thermosensitive hydrogels**	Poloxamer 188 (1%) and poloxamer 407 (24%).	Hydrogels were prepared by the cold swelling method. MTT assay indicated the safety of the developed formulation and its tendency to enhance the proliferation of human fibroblasts. The medicated hydrogels enhanced the wound healing of the induced FTW in rats. New epithelium and connective tissues were formed in an organized and complete manner. The regenerated epithelium and connective tissue arrangement of the wounds were more standardized and complete than in the control group.	[109]
**Films**	Bacterial cellulose, alginate and gelatin	The developed films were considered as thin films with excellent bioadhesive properties as well as enhanced fluid uptake capability up to 700%. Good antibacterial activities against *E. coli* and *S. aureus* were achieved. The prepared films were non-cytotoxic on human keratinocyte. Additionally, the proliferated cellular extensions were such as the vivo tissues.	[110]
**Scaffolds**	Sodium alginate and collagen	The results of in vivo wound healing after 14 days using Hematoxylin and Eosin staining indicated that curcumin-loaded scaffold was more potent than the untreated control.	[111]
**Sponge**	Cellulose sponge, β-cyclodextrin and chitosan	The poor aqueous solubility of curcumin was improved by its inclusion in β-cyclodextrin and formation of inclusion complex. The cellulose sponges had a porous structure and its mechanical properties were improved by inclusion of both the cyclodextrin complex and chitosan. The presence of chitosan enhanced the antibacterial properties of the formulation. The sponge was compatible with NCTC L929 and NHDF cells. Finally, it can be used as wound dressing especially in chronic wound.	[112]
**Liposomes**	Pluronic F127 augmented liposomes	The influence of adding Pluronic F127 to the curcumin-loaded liposomes was investigated on human keratinocyte cell line. Results of MTT test revealed the safety of the formulation. Pluronic F127 augmented liposomes improved the cell migration as well as the expression of both the nuclear factor erythroid-related factor 2 and kelch, such as erythroid cell-derived protein 1 compared to the pure curcumin powder and the unmedicated liposomes, hence offering a promising formulation for better wound healing.	[113]
**Nanoparticles**	Tetramethyl orthosilicate and chitosan	The fabricated nanoparticles inhibited the growth of MRSA in vitro as well as in an in vivo murine burn model. Accelerated wound healing with better formation of granulation tissues as well as collagen was observed in curcumin-loaded nanoparticle-treated group respective to the sulfadiazine-treated group. Less tissue loss post-injury procedure (early stages) was detected in curcumin-loaded nanoparticles group compared to the sulfadiazine-group. The obtained results offered the designed nanoparticles as a promising platform for the treatment of burns and suggested the reestimation of the conventional burn therapy; sulfadiazine.	[114]
**Mesoporous silica particles**	Mesoporous silica powder	Curcumin-loaded mesoporous silica nanoformulation was prepared by simply mixing curcumin solution along with mesoporous silica powder under heating at 50 °C. Round excision wounds were created on the back of the rats which were divided into two groups; one group was treated with curcumin-loaded mesoporous silica nanoformulation and the other group with sulfadiazine. Curcumin-treated group showed better improvements in the healing process which was endorsed to the anti-inflammatory effect of the formulation as well as its ability to enhance the angiogenesis process, epithelization and collagen synthesis.	[115]
**Mixed polymeric micelles**	Chitosan, sodium alginate, maltodextrin, Pluronic^®^ F127, Pluronic^®^ P123, and Tween^®^ 80	Curcumin-loaded mixed micelles were prepared using thin film hydration technique. Excellent wound healing was observed in the group of animals treated using the mixed micelles which was endorsed to the antioxidant and anti-inflammatory effect of curcumin as well as to the presence of both chitosan and sodium alginate which are known to enhance the wound healing process due to protection of the wounds from bacterial infections. The proposed formulation is considered a safe, biocompatible carrier for wound healing purposes.	[116]
**Nanoemulgel**	Labrafac PG (oil), Tween^®^ 80 (surfactant), and PEG-400 (co-surfactant)	The nanoemulgel was prepared using ultrasonic emulsification method. The optimized nanoemulsion was 50 nm in size using the least amount of the surfactant concentration. Nanoemulgels were prepared by the incorporation of the selected nanoemulsion into a 0.5% Carbopol^®^ 940 hydrogel matrix for topical application. The designed nanoemulgel possessed excellent skin penetrability and promising wound healing capabilities in Wistar rats.	[117]
**Nanoemulsion**	Clove oil (oil), Tween^®^ 80 (surfactant), and PEG-400 (co-surfactant)	Histopathological examination of the wounds indicated the safety and the non-toxicity of the applied nanoemulsion due to the lack of the inflammatory cells. The selected formulation enhanced the wound healing in rats due to the enhancement in the proliferation of the epithelial cell proliferation. Additionally, the selected formulation showed excellent anti-inflammatory effects for the healing of edema in carrageenan-induced rat paw edema model.	[118]
**Carbon dots**	Carbon dots, protease-responsive hydrogel	The formulation of curcumin in the form of carbon dots improved the solubility and stability of the free curcumin. The enhanced proliferative, proangiogenic and antibacterial activity of carbon dots made them a good choice for wound healing applications. A cross-linker, protease-responsive hydrogel was used to sustain the drug release. After application of the developed formulation on a skin excision model, it was apparent that the carbon dots supported with protease responsive hydrogel showed faster wound contraction with enhanced angiogenesis and full formation of the epithelium.	[119]
**Nanofibrous scaffolds**	Cellulose acetate and 10% poly (ε-caprolactone)	The nanofibrous scaffolds were fabricated by electrospinning. Curcumin possessed bi-functional role as a drug and as hydrophilicity enhancing agent due to the hydrogen bonds formed between the components which in turn enhanced the swelling capacity to around 700 or 950% according to the % of the added curcumin. The fabricated medicated scaffolds boosted the expression of actin in fibroblasts than the unmedicated ones.	[120]
**Nanofibrous mats**	Gelatin	The nanofibrous mats were fabricated by electrospinning. Full-thickness wounds were created on the backs of Sprague Dawley male rats. The wounds in one group of the animals were treated with curcumin/gelatin mats and compared to a group of animals treated with gelatin mats lacking the addition of curcumin. Results showed significantly faster wound closure on 7th, 11th and 15th day post-wounding with curcumin/gelatin nanofibrous mats treated groups. This enhanced wound closure might be attributed to the complete reepithelization and formation of well-developed epidermis.	[121]
**Nanohydrogel**	Deformable liposomes-in-chitosan hydrogels Lipoid S100 was used for the preparation of liposomes	The developed nanohydrogel combined the wound healing properties of both curcumin and chitosan. The positively charged liposomes prepared using stearylamine as a positive charge inducer provided better bioadhesion as well as enhanced and sustained penetration through full-thickness human skin compared to the neutral and anionic liposomes.	[122]

## Data Availability

The data presented in this study are available in the article.

## Abbreviations

FTW	full thickness wound
DM	diabetes mellitus
ROS	reactive oxygen species
ECM	extracellular matrix
